# Calculating the Social Rhythm Metric (SRM) and Examining Its Use in Interpersonal Social Rhythm Therapy (IPSRT) in a Healthy Population Study

**DOI:** 10.3390/bs4030265

**Published:** 2014-08-06

**Authors:** Theun Pieter van Tienoven, Joeri Minnen, Sarah Daniels, Djiwo Weenas, Anke Raaijmakers, Ignace Glorieux

**Affiliations:** 1 Research Group TOR, Sociology Department, Vrije Universiteit Brussel, Pleinlaan 2, 1050 Brussels, Belgium; E-Mails: joeri.minnen@vub.ac.be (J.M.); sarah.daniels@vub.ac.be (S.D.); djiwo.weenas@vub.ac.be (D.W.); ignace.gloriex@vub.ac.be (I.G.); 2 Pediatrics Department, UZ Leuven, Herestraat 49, 3000 Leuven, Belgium; E-Mail: anke.raaijmakers@uzleuven.be

**Keywords:** bipolar disorder, cognitive behavioral therapy, interpersonal psychotherapy, social rhythms, GHQ-12, time-diary

## Abstract

In psychiatry, the social zeitgeber theory argues that social life provides important social cues that entrain circadian rhythms. Disturbance of these social cues might lead do dis-entrainment of circadian rhythms and evoke somatic symptoms that increase the risk of mood disorders. In preventing and treating patients with bipolar disorders, the Interpersonal and Social Rhythm Therapy (IPSRT) relies on the Social Rhythm Metric (SRM) to (re)establish patients’ social cues and an re-entrain circadian rhythms. Since the SRM quantifies social rhythms that are derived from a patient’s interaction with a social environment, this contribution (a) calculates the SRM of the social environment of a representative healthy population study (n = 1249), (b) evaluates the robustness of the SRM as a quantifier of social rhythms by matching the scores of the pilot study, revealing the near absence of variance across population characteristics and investigation months—circadian rhythms need to be entrained for every month and for everyone—and (c) examines its use in IPSRT by relating high SRM-scores to lower psychological distress (*p* = 0.004) and low SRM-scores to higher social and emotional dysfunction (*p* = 0.018).

## 1. Introduction

Cyclic and repetitive time is essential for the experience of stability and sameness in everyday life [1]. This ism is subjacent to psychotherapy known as Interpersonal and Social Rhythm Therapy (IPSRT) targeting the prevention and recovery of depression in patients with bipolar disorder [2,3,4]. If repetitive behaviors create the appearance of structure and permanence in the flow of life, the corollary assumption equally holds true that a disturbance hereof in the form of a life event undermines this sense of stability. Following the logic of the social zeitgeber hypothesis [5] a life event changes social prompts (or social zeitgebers), leading to a change in the stability of social rhythms, which in turn changes the stability of biological rhythms affecting somatic symptoms and ultimately causing manic and depressive episodes.

The social zeitgeber hypothesis is based on two assumptions: (1) disturbances in circadian rhythms shown by many physiological processes are important in the pathophysiology of mood disorders and (2) both physical and social cues entrain human circadian rhythms. Circadian literally implies that it concerns rhythms that follow only approximately 24 hours and several physiological processes have been shown to “free-run” at a slightly longer period [6,7]. Many researchers suspect the suprachiasmatic nucleus in the anterior of the hypothalamus to function as the internal pacemaker for these processes and consider light as the main physical zeitgeber that entrains this master clock to keep up with the 24 hours. It follows that the light/dark cycle and the concurring sleep/wake cycle is the most overt manifestation of the human circadian system, of which the association of sleep-disturbance and depression are the most profoundly studied. As a result, circadian hypotheses of depression are often related to sleep disorders. Hence therapies for depression include light therapy, “wake therapy”, or therapies to increase REM latency (electroconvulsive therapy, transcranial magnetic stimulation) [8].

Although light is an important physical cue that entrains circadian rhythms, humans have become increasingly detached from daylight schedules in their behavior and social cues have recently gained importance to keep circadian rhythms on track. Among these social cues are sleeping habit, rigidity of eating times and other routines arising form social interaction and being shaped by familial, social and occupational roles [9]. These social rhythms not only structure the day in terms of sequences, coordination and synchronization of activities, but also serve as (social) zeitgebers that keep daily circadian rhythms entrained to 24 hours [5,6,7,9,10,11]. A loss of or change in these social zeitgebers may derail or disentrain circadian rhythms [9] and thus induce somatic symptoms that may lead to a (major) depressive episode [12,13]. Therefore, a quantification of these social rhythms could be of use for future investigation of the aetiology of depression or cognitive behavior therapy that includes the attempt to (re)establish daily routines of activities [9].

The Social Rhythm Metric (SRM) is such a method for quantification [9,10]. It gives a score based on the timing of 15 specific and 2 built-in activities that are thought to constitute an individual’s social rhythm. If the timing of an activity that occurs at least three times a week is within 45 minutes of the average or “habitual time” it is considered a “hit” for daily routine. The total number of hits of these activities divided by the total number of activities occurring at least three times a week gives the SRM-score. A higher SRM-score was found to relate to subjective better sleep, higher morning alertness and a deeper nocturnal temperature trough [11], whereas lower SRM-scores correlated with higher reports of depressive symptoms.

By including the SRM in its behavioral psychotherapy for affective disorder, the Interpersonal and Social Rhythm Therapy (IPSRT) attempts to reduce the number and severity of patients’ interpersonally or socially based stressors (which cause reductions in sleep and appetite and marked changes in daily routines and social rhythms) and increase the stability of social rhythms and patients’ watchfulness to this social stableness [2]. IPSRT serves as a complement to the use of pharmacotherapy in treating bipolar I disorders and either as a stand-alone treatment or along with pharmacotherapy in treating bipolar II disorders [2]. Within the four-phase treatment of IPSRT, the SRM instrument is used in the intermediate phase (*posterior* to the initial phase) to regularize the patient’s social rhythms and in the continuation phase to help patients maintain their daily routine (*prior* to the final phase) [2,3,4]. Reported results include findings that patients subjected to IPRST in the acute treatment phase experienced longer survival time without relapse and were more likely to stay well for full 2 years of the preventive maintenance phase [14] and that IPSRT plays an important role in establishing and reproducing daily activities such that it enables successful return to work [15]. This confirms the social zeitgeber hypothesis, at least with respect to the relationship between social rhythms and risk of new episodes.

What this contribution is concerned with, is the validity of the SRM-instrument itself. Whether it is the original SRM [9] or the adapted SRM-II [2], SRM-II-5 (adult version with five activities) or SRM-A (adult version with 8 activities) [3] its use in IPSRT turned the SRM-instrument into a means instead of an end and, hence, almost no SRM-scores are reported except in the initial work introducing the methodology [9]. The output is measured in terms of efficiency of the treatment, whereas the question may arise how to conclude patients’ daily routine or interpret patients’ SRM-score? What is the comparative SRM-score of the average daily routine of a healthy population in which patients’ need to reintegrate and/or maintain themselves? (Note that throughout this contribution we use the word “healthy” to contrast a normal population with patients eligible for IPSRT.) How does one know whether patients’ daily activities are not “over-routinized” or still lack sufficient structure? The only “normal” scores we found are the ones of a small sample repeated pilot study of normal control persons.

Conducting a population study to determine a healthy population’s daily routine and validate the instrument on this scale is probably impracticable. However, there are *existing* data that make it possible to do so. Using such data we will evaluate the robustness of the SRM as a measure of daily routine and its use in IPSRT by the following four conditions: the population SRM-score should (1) approximate the scores of the normal control pilot study, (2) hardly vary over the research months and (3) across socio-economic population characteristics (since social entrainment of circadian rhythms should hold approximately equally for every month and for everyone) and (4) relate in like manner to self-reported general health.

## 2. Methods

### 2.1. Data

In selecting the most appropriate database, we used the following conditions: (1) it should be a population study, (2) that is representative for modern societies, (3) that is spread as well as possible over a whole calendric year to include seasonal fluctuations, (4) that contains time-diary information of a whole week (since the SRM is based on a whole week) and (5) that has information on self-reported general health (to relate with the SRM score). Only a few 7-day diary studies exist: the UK 1974–1975 study, the Dutch 5-yearly 1975–2011 studies and the Flemish 1999 and 2004 studies. Although the Dutch data might provide more up to date figures, these data cover only two weeks in October whereas the Flemish data cover April to November and are the only data including an item scale on self-reported general health. Nonetheless, we realize that exclusion of winter months is unfortunate because of their cause of Seasonal Affected Disorders (SAD).

For this study we thus use time-diary data of a sample of the Flemish population of 2004 (abbrev. TOR04), collected by the Research Group TOR of the Vrije Universiteit Brussel and representative for a modern high-income society. (Flanders is the Dutch speaking part of Belgium and has over 6 million inhabitants, or about 60 per cent of the Belgian population. In 2004 the GDP per capita at purchasing power was 23 per cent above the EU average.) Data are weighted by gender, age and educational level to correct for differences with the population register. The sample is limited to 24- to 65-year-olds. Students and respondents still living with their parents are excluded, because we expect their living conditions to be very different from the rest of the population. This brings the total sample size at n = 1249. Table 1 presents the demographic characteristics of the sample.

**Table 1:** Demographic characteristics of sample (n = 1249).

Characteristic	Categories	Percentage
**Gender**	Female	51.3%
	Male	48.7%
**Age**	25–39 years	33.5%
	40–54 years	45.2%
	55–65 years	21.2%
**Education**	Low	29.0%
	Medium	35.6%
	High	35.4%
**Employment**	Full time	54.4%
	Part-time	19.0%
	Unemployed	16.7%
	Early retirement	9.8%
**Family situation**	Alone	12.9%
	Single parent	5.4%
	Partner no kids	24.3%
	Partner and kids	57.3%

Respondents in the TOR04 study kept a time-diary for 7 consecutive days. Each entry in the time-diary existed of a primary activity code (what did you do?), a secondary activity code (what else did you do?), the beginning and ending time of the activity, and some contextual information (location, presence of others, motivation) (see Figure S1 in supplementary). Activity codes were derived from a pre-coded list of activities that was distributed together with the time diary. Whenever one of these elements changed, a new entry had to be made. Additionally, respondents completed a pre- and post-questionnaire, which included the 12-item version of the General Health Questionnaire (GHQ-12) [16,17,18].

### 2.2. Method of Conversion

In comparison to time diary studies respondents involved in a SRM study complete a diary sheet every evening for at least a whole week indicating the starting time of the first occurrence of a list of 15 fixed and 2 variable activities (the latter are chosen after consultation of their therapist). Additionally they indicate the presence of others (if any) for each of their activities [9,10]. Table 2 shows how information from the time-use data is converted to meet the list of activities of the SRM diary sheet.

**Table 2:** Overview conversion activities form Social Rhythm Metric (SRM) diary sheet to TOR04 diary.

Activity number	Description in SRM	Conversion to TOR04
1	Out of bed	First non-sleep activity not followed by a new sleep activity
2	First contact (in person or by phone) with another person	First activity with indication of personal interaction or first phone activity
3	Have morning beverage	First drinking activity between 4 am and 11 am
4	Have breakfast	First eating activity between 4 am and 11 am
5	Go outside for the first time	First activity involving change of location form *at home* to *not at home*
6	Start work, school, housework, volunteer activities, child or family care	First work, school, housework, volunteer, childcare or family care activity
7	Have lunch	First eating activity between 11 am and 4 pm
8	Take afternoon nap	First nap/rest activity after 12 am
9	Have dinner	First eating activity between 4 pm and 9 pm
10	Physical exercise	First sport, walking, cycling, going to swimming pool activity
11	Have an evening snack/beverage	First eating/drinking activity between 9 pm and 4 am
12	Watch evening TV news program	First activity watching TV (all programs) at any time of the day
13	Watch another TV program	First activity doing some reading (book, magazine, newspaper, advertising brochures) at any time of the day
14	Activity A:	First activity done out of *duty*
15	Activity B:	First activity done out of *pleasure*
16	Return home (last time)	First activity *at home* after which location does not change anymore
17	Go to bed	First 5 h sleeping episode

Some minor deviations had to be overcome and were mostly due to the high level of detailed activities of the SRM and the continuous registration method of TOR04. First, note that time-diary methodology exists of a continuous registration of activities, that is, respondents do not write down “I got up at 7:00” but simply register their next activity (e.g., showering, making breakfast). To deduce the time respondents gets up, we, therefore, identified the moment at which a respondents registered their first non-sleeping activity followed by another non-sleeping activity to exclude respondents that got up to go to the bathroom and got back into bed hereafter.

Second, to account for the timing of meals, we divided the day into time-slots in which the different meals are generally consumed. The timing of someone’s breakfast is then captured as the first eating activity between 4:00 and 11:00, lunch as the first eating activity between 11:00 and 16:00, dinner as the first eating activity between 16:00 and 21:00, and evening snack/drink as the first eating and/or drinking activity between 21:00 and 4:00.

Third, the distinction between watching TV news program and other TV programs could not be made based on the activity codes in TOR04, so we included the first activity watching any TV program and added the first activity any reading is done (including newspaper).

Fourth, we replaced items A and B by the first activity performed out of duty and the first activity performed out of pleasure. By the former we mean an activity that one is required to do, by the latter an activity from which one derives enjoyment [19]. Note that these are subjective judgments made by respondents themselves.

### 2.3. Statistical Method

For each activity listed in Table 2 that occurs more or equal to three times a week a ± 45-min range from its non-outlier mean is used to calculate a possible “hit” if the occurrence of the activity falls within this range. To correct for unforeseen events that might disrupt regular social rhythms, we divided the sample in two by the respondent’s indication whether the registration week was special for any reason or not (as indicated in a post-questionnaire). Among the most mentioned reasons, we find being hospitalized or on vacation.

Next, the variance of the replicated SRM-score across the population is analyzed by a multiple classification analysis (MCA) to predict the adjusted means of SRM score. We include gender, age (25–39, 40–54, 55–65 years), education (low, medium, high), employment (full time, part-time, unemployed, early retirement) and family situation (alone, single parent, partner no kids, partner and kids) as population characteristics.

Then, a multivariate linear regression with separate analyses for the non-special and special week group and controlling for the aforementioned population characteristics, relates the SRM score to an indicator of self-reported general health. For statistical reasons the SRM is transformed into dummy variables: low ( ≤ x − 1 s.d.) and high ( ≥ x + 1 s.d.) *vs.* medium SRM-score. Self-reported general health is measured by the 12-item version of the General Health Questionnaire (GHQ-12) [16,17,18] using a five point Likert scale ranging from total disagreement to total agreement. The item “I have been thinking of myself as a worthless person” has been omitted because of too many missing values. The item “I have been feeling reasonably happy, all things considered” has been omitted after reliability test of the obtained components. A principal component analysis, then, yields a two-component solution (51.4% of the variance is explained) that concurs with earlier solutions [20]. The first component captures psychological distress (Eigen value = 3.873, Cronbach’s alpha = 0.757) the second social and emotional dysfunction (Eigen value = 1.268, Cronbach’s alpha = 0.691) (see Table S1 in supplementary). We converted the Z-scores of both scales to T-score for ease of interpretation (psychological distress: non-outlier min = 27.0, max = 71.6; social and emotional dysfunction: non-outlier min = 30.1, max = 69.9). 

All calculations and analyses are performed in IBM^®^ SPSS^®^ Statistics Version 22. 

## 3. Results

**Figure 1:**
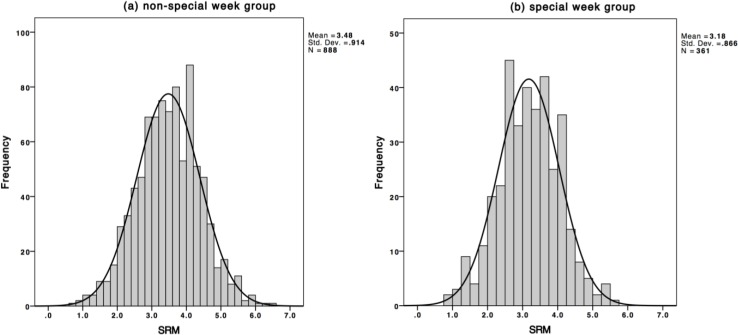
Frequency histogram of population SRM scores, together with equivalent normal distribution for (**a**) non-special week group and (**b**) special week group.

Figure 1 illustrates the frequency distribution for both groups (correlation between groups and SRM, *r* = − 0.149, two-tailed *p* < 0.001). A normal distribution with appropriate parameters (non-special week, 3.48 ± 0.914 [x ± s.d.]; special week, 3.18 ± 0.866; means differ significantly, *t* = 5.329, *p* < 0.001) was generated and superimposed on the actual frequency histogram (Kolmogorov-Smirnov, non-special week, *D* = 0.026, *p* = 0.150; special week, *D* = 0.027, *p* = 0.200).

**Figure 2:**
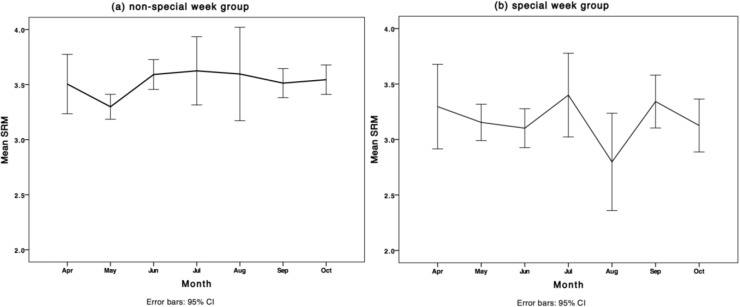
Population mean SRM-score per month and 95% CI of mean for (**a**) non-special week group and (**b**) special week group.

Figure 2 gives the population mean SRM-score per month included in the study separately for both groups. The error bars represent the 95% CI of the mean. November is excluded because of too little cases. Only the mean SRM-scores of the non-special week group are significantly different over the months (non-special week, *F* = 2.017, *p* = 0.024; special week, *F* = 1.068, *p* = 0.382), although the effect size is small (*η^2^* < 0.1) [21,22].

Table 3 contains a Multiple Classification Analysis (MCA) presenting both unadjusted (bivariate) and adjusted (controlled for the population characteristics in the model) predicted means of the SRM-score for both groups. The unadjusted predicted means for the non-special week group only significantly differ by age, employment and family situation; the adjusted means only by age and family situation. Co-habiting with partner and kids, early retirement and 55- to 65-year-olds yield the highest SRM-scores, although again effect size (*η^2^* < 0.1) and explained variance (*Adj. R^2^* = 0.079) are low. For the special week group none of the predicted means differed significantly.

Descriptive information of respondents with the lowest (<2.0; n = 8) and highest (>4.5; n = 7) SRM-scores did not reveal much evident characteristics that contributed to these differences (results not shown). Although the lowest SRM-scores include almost only respondents living alone and the highest SRM-scores only 55–65-year-old males, the number of respondents is too low for statistical inferences. Shifting the boundaries of lowest and highest SRM-scores did not create more homogeneity with regard to these characteristics.

Table 4 presents the results for the multivariate linear regression models. High SRM-scores are significantly negatively associated with a lower self-reported psychological distress and low SRM-scores with higher social and emotional dysfunction for the non-special week group. No significant relationships are found for the special week group.

**Table 3:** Multiple classification analysis (MCA) of the SRM-scores in Flemish population study (n = 1249).

Characteristic	Categories	Non-special week (n = 888)		Special week (n = 361)	
		Predicted mean		Predicted mean	
		*Unadjusted*	*Adjusted for factors*	*Unadjusted*	*Adjusted for factors*
**Gender**	Female	3.49	3.49	3.23	3.22
	Male	3.46	3.46	3.12	3.13
**Age**	25–39 years	3.38	3.40	3.09	3.08
	40–54 years	3.36	3.34	3.15	3.14
	55–65 years	3.83	3.85	3.41	3.46
		***	***		
**Education**	Low	3.46	3.47	3.14	3.13
	Medium	3.58	3.53	3.19	3.15
	High	3.40	3.44	3.19	3.23
**Employment**	Full time	3.39	3.45	3.12	3.16
	Part-time	3.41	3.40	3.22	3.21
	Unemployed	3.57	3.52	3.24	3.20
	Early retirement	3.89	3.66	3.35	3.17
		***			
**Familysituation**	Alone	3.18	3.09	2.98	2.92
	Single parent	3.31	3.37	3.09	3.13
	Partner no kids	3.53	3.37	3.28	3.21
	Partner and kids	3.54	3.63	3.18	3.22
		***	***		

Note: * *p* < 0.05, ** *p* < 0.010, *** *p* < 0.001 for within category differences (two-tailed).

**Table 4:** Multivariate linear regression models of replicated SRM score predicting self-reported general health (T-scores) in a population study.

	Coefficients ^a^				
**Psychological distress—non-special week (n = 888)**	*b*	*s.e.*	*bèta*	*sig.*	*ρ_xy·z_*
Constant	49.353	1.112			
Low SRM (*ref. = Medium*)	0.967	0.983	0.034		0.033
High SRM (*ref. = Medium*)	−2.570	0.896	−0.099	**	−0.093
Total model variance explained *Adjusted R^2^* = 0.010					
**Psychological distress—special week (n = 361)**	*b*	*s.e.*	*bèta*	*sig.*	*ρ_xy·z_*
Constant	48.521	2.030			
Low SRM (*ref. = Medium*)	1.040	1.417	0.041		0.039
High SRM (*ref. = Medium*)	0.624	1.935	0.018		0.017
Total model variance explained *Adjusted R^2^* > 0.001					
**Social and emotional dysfunction—non-special week (n = 888)**	*b*	*s.e.*	*bèta*	*sig.*	*ρ_xy·z_*
Constant	49.537	1.060			
Low SRM (*ref. = Medium*)	2.218	0.937	0.082	*	0.078
High SRM (*ref. = Medium*)	−0.613	0.854	−0.024		−0.024
Total model variance explained *Adjusted R^2^* = 0.030					
**Social and emotional dysfunction—special week (n = 361)**	*b*	*s.e.*	*bèta*	*sig.*	*ρ_xy·z_*
Constant	51.780	2.181			
Low SRM (*ref. = Medium*)	−0.808	1.522	−0.029		−0.028
High SRM (*ref. = Medium*)	−0.985	2.079	−0.025		−0.025
Total model variance explained *Adjusted R^2^* = 0.026					

Note:^ a^ All coefficients are controlled for gender, age group, education, employment situation and family situation.

## 4. Discussion

Physical cues entrain the biological processes that make up circadian rhythms and a disruption hereof is found to relate to mood disorders. To this, the social zeitgeber hypothesis adds that social cues (a synonym for daily routine of rigid eating times, sleeping habits, *etc.*) also entrain these processes. Disruptions of daily routines due to life events disentrain the underlying physiological processes of circadian rhythms and may result in depression. In preventing or treating bipolar disorders an instrument quantifying these daily routines serves well for a therapist to help patients recognize and live by their daily routines. The IPSRT does so by using the SRM and results provide evidence for the social zeitgeber hypothesis.

The question that arises is how to judge this quantification of daily routines? How to interpret a score that lies somewhere between uniqueness (minimum score) in everyday life and total predictability (maximum score) of everyday life? What is the average score of daily routine of a healthy population? In other words: what is a comparative SRM-score?

The problem relates to the need for a population study, which often exceeds available resources. As far as we know, only a small-scale pilot study (n = 50) reports SRM-scores of healthy subjects. Some additional insights might come from time-diary population studies among which there exist a few unique studies that have time-diary data for 7 consecutive days. Using the Flemish 2004 time-diary study (n = 1249) we replicated the SRM and examined its robustness based four propositions: (1) approximate the SRM-score of the pilot study (2) have stable dispersion of the SRM-scores over the different investigation months, (3) and across population characteristics, and finally and most importantly (4) relate the SRM-scores to self-reported general health.

First, the mean population SRM-score of the non-special week group of 3.48 is very close to the results of the repeated pilot study of normal control subjects (week 1, 3.44 ± 0.91 [x ± s.d.], week 2, 3.55 ± 0.93). In line with the idea behind the SRM the mean for special week group is somewhat lower (3.18). Second, the mean SRM-scores did vary significantly over the months for the non-special week group, but the effect size was negligibly small. Third, the population mean of the non-special week group varies slightly with age (the elderly having the highest SRM-score) and family situation (the more household members present, the higher the SRM-score), but again effect size and explained variance are negligible. This demonstrates the robustness of the SRM: it quantifies someone’s daily rhythm and yields comparable results without having to take into account the month of investigation or population characteristics.

Fourth, only for the non-special group a higher SRM-score significantly relates to lower psychological distress and a lower SRM-score to higher social and emotional dysfunction. Nonetheless, all models have low levels of explained variance (low *Adj. R^2^*) and low partial correlation coefficients (low *ρ_xy·z_*) indicating that there are mainly other factors than population characteristics or (disturbance of) social rhythms that count for the variance in self-reported general health.

Despite the fact that we used data in which subjects for whom the SRM has initially been developed are highly unlikely to be included, we do confirm the SRM as a robust indicator of social rhythms. We also find results that confirm the correlation between SRM-score and psychological distress and social and emotional dysfunction as it is assumed for its use in IPSRT, although figures are rather lean.

Two final remarks need to be made. First, in the SRM all activities are treated equally, though we might argue not having a snack or physical exercise or not watching TV will have less impact on someone’s social rhythm than not sleeping for a whole night. Calculating the SRM by pre-assigning weights to the items included might overcome this issue, but since this involves making many assumptions such a procedure falls beyond the scope of this contribution. This issue is probably also one of the reasons why multiple versions of the original SRM exist [3,4].

Secondly, we need to mention that in IPSRT patients keep a time sheet for several weeks in order to keep track of the progress of recovering their daily rhythm. Our data only include one week per respondent and, although the week is argued to be one of the most important temporal frameworks for the organization of daily life [1], we do realize that activities that transcend this weekly structure, like irregular working arrangements such as shift work or being on call or like receiving monthly salary that will affect shopping activities and thus might cause SRM-scores to vary across weeks, are not taken into account. 

## 5. Conclusions

The IPSRT deals with individual patients and uses the SRM to focus on the recognition and (re)establishment of their personal daily routines. In terms of treatment and outcomes and according to the social zeitgeber hypothesis, the (re)establishment of daily routines should result in social rhythmicity of everyday life that restores the social zeitgebers that entrain circadian rhythms and thus divert the risk of relapse. Examining (the use of) the SRM by a population study and finding a score for a normal social rhythm of 3.5 might in first instance seem irrelevant with respect to IPSRT (as the degree of daily routine needed undoubtedly varies from patient to patient) although we argue otherwise.

Every individual is part of a social group: a family, a neighborhood or a society as a whole, and every individual has to function within the habits of this group (and will be deemed to conform to this). Having a comparative value that reflects the daily routine of a representative healthy population will help to interpret the patient’s (need for) daily routine and judge the perspective of a patient when fully (re)integrated in everyday social life. The IPSRT might be considered successful if patients remain without new effective episodes for a long time but what if this is requires a daily routine at an SRM-score of, say, 5.2? This indicates that patients might need a social environment that is characterized by unrealistic stability and be vulnerable for realistic coincides that might affect or shift their daily routines. Such patients might still need special attention when reducing therapy.

We, by no means, recommend targeting a patient’s daily routine to match the daily routine of the normal population, but we do aim to provide some social context that allows the interpretation of a patient’s daily rhythm, because following a *social* rhythm ultimately implies functioning in a *social* environment that has its own *social* rules, including those relating to daily routines.

## Supplementary Files

Supplementary File 1

## References

[1] Weigert A.J. (1981). Chapter 5: Time in everyday life. Sociology of Everyday Life.

[2] Frank E. (2007). Interpersonal and social rhythm therapy: A means of improving depression and preventing relapse in bipolar disorder. J. Clin. Psychol..

[3] Frank E., Maggi L., Miniati M., Benvenuti A. (2009). The rationale for combining interpersonal and social rhythm therapy (ipsrt) and pahrmacotherapy for the treatment of bipolar disorders. Clin. Neuropsychiatry.

[4] Frank E., Swartz H.A., Kupfer D.J. (2000). Interpersonal and social rhythm therapy: Managing the chaos of bipolar disorder. Biol. Psychiatry.

[5] Grandin L.D., Alloy L.B., Abramson L.Y. (2006). The social zeitgeber theory, circadian rhythms, and mood disorders: Review and evaluation. Clin. Psychol. Rev..

[6] Aschoff J., Fatranska M., Giedke H., Doerr P., Stamm D., Wiser H. (1971). Human circadian rhythms in continous darkness: Entrainment by social cues. Science.

[7] Wever R. (1975). Autonomous circadian rhythms in man. Naturwissenschaften.

[8] Germain A., Kupfer D.J. (2008). Circadian rhythm disturbances in depression. Hum. Psychopharmacol. Clin. Exp..

[9] Monk T.H., Flaherty J.F., Frank E., Hoskinson K., Kupfer D.J. (1990). The social rhythm metric. An instrument to quantify the daily rhythms of life. J. Nerv. Ment. Dis..

[10] Monk T.H., Kupfer D.J., Frank E., Ritentour A.M. (1991). The social rhythm metric (srm): Measuring daily social rhythms over 12 weeks. Psychiatry Res..

[11] Monk T.H., Petrie S.R., Hayes A.J., Kupfer D.J. (1994). Regularity of daily life in relation to personality, age, gender, sleep quality and circadian rhythms. J. Sleep Res..

[12] Ehlers C.L., Frank E., Kupfer D.J. (1988). Social zeitgebers and biological rhythms: A unified approach to understanding the etiology of depression. Arch. Gen. Psychiatry.

[13] Ehlers C.L., Kupfer D.J., Frank E., Monk T.H. (1993). Biological rythms and depression: The role of zeitgebers and zeitstörers. Depression.

[14] Frank E., Kupfer D.J., Thase M.E., Mallinger A.G., Swartz H.A., Fagiolini A.M., Grochocinski V., Houck P., Scott J., Thompson W. (2005). Two-year outcomes for interpersonal and social rhythm therapy in individuals with bipolar i disorder. Arch. Gen. Psychiatry.

[15] Frank E., Soreca I., Swartz H.A., Fagiolini A.M., Mallinger A.G., Thase M.E., Grochocinski V.J., Houck P.R., Kupfer D.J. (2008). The role of interpersonal and social rhythm therapy in improving occupational functioning in patients with bipolar i disorder. Am. J. Psychiatry.

[16] Goldberg D.P., Williams P. (1988). A User's Guide to the GHQ.

[17] Goldberg D.P., Gater R., Sartorius N., Ustun T.B., Piccinelli M., Gureje O., Rutter C. (1997). The validity of two versions of the ghq in the who study of mental illness in general health care. Psychol. Med..

[18] Goldberg D.P. (1972). The Detection of Psychiatric Illness by Questionnaire.

[19] Glorieux I. (1990). Social interaction and the social meanings of action: A time-budget approach. Soc. Indic. Res..

[20] Werneke U., Goldberg D.P., Yalcin I., Üstün B.T. (2000). The stability of the factor structure of the general health questionnaire. Psychol. Med..

[21] Cohen J. (1988). Statistical Power Analysis for the Behavior Sciences.

[22] Cohen J. (1992). A power primer. Psychol. Bull..

