# Supplementary material for: Calculating the Social Rhythm Metric (SRM) and Examining Its Use in Interpersonal Social Rhythm Therapy (IPSRT) in a Healthy Population Study
Source: Behav Sci (Basel). 2014 Aug 6;4(3):265–77. doi: 10.3390/bs4030265 (PMC4219261; doi:10.3390/bs4030265)
Supplement: Supplementary File 1 [file behavsci-04-00265-s001.pdf]

Supplemental Materials

Figure S1. Completed diary page (translated from Dutch). For each new activity all questions (A to J) had to be answered.

A. Activity: ***(give activity code)***

431 (=eating cold foot)

B. Secondary activity: ***(use activity code)***

920 (=reading newspaper)

C. Starting time: 7h30m

D. Ending time: 7h52m

E. New day: ***(give dd/mm/yy)***

21/06/ 04

F. Location **(encircle 1 answer):**

1. At home (also student dorm or holiday home)
2. At work (not at home)
3. At someone else's place
4. Somewhere else

G. In case of transport, by which means?
(encircle – multiple answers possible)

1. By foot
2. By bike
3. By moped or scooter
4. By motorcycle
5. By car
6. By public transport (bus, tram, metro,train)
7. By other means

Comments:

H. Presence of others:
(encircle 1 answer)

1. Nobody
2. Yes, there was someone else present
3. Yes, I did the activity with someone else

I. Spoken with:
(encircle – multiple answers possible)

1. Nobody
2. Spouse, partner, …
3. (Own) children with whom you live together
4. Parents with whom you live together
5. Siblings with whom you live together
6. Other members of the household
7. Non-resident family members
8. Neighbours
9. Colleagues, fellow students, …
10. Friends, acquaintances, …
11. People that provide a service
12. People to whom you provide a service
13. Someone unknown
14. Others : ……………………

J. I did this activity because …
(preferably encircle only 1 answer)

1. I am required or obliged
2. To please others, or out of feelings of duty
3. Out of necessity, because it is required before doing something else, because there is no other
4. Of the pleasure I derive form doing it

Table S1. Varimax rotated component matrix of principal component analysis (sorted) of GHQ-12.

**Item** [a,b]	**Mental distress**	**Mental stability**
I lost much sleep over worry	**0.783**	−0.003
I felt constantly under constrain	**0.779**	0.030
I have been feeling unhappy and depressed	**0.650**	−0.413
I have been loosing confidence in myself	**0.533**	−0.469
I felt I couldn't overcome my difficulties	**0.516**	−0.474
I felt capable of making decisions about things	−0.135	**0.709**
I have been able to face up to my problems	0.145	**0.706**
I felt I was playing a useful part in things	−0.228	**0.635**
I have been able to enjoy my normal day-to-day activities	−0.473	**0.470**
I have been able to concentrate on what I am doing	−0.417	**0.466**
Eigen value	3.873	1.268
Cronbach's Alpha	0.757	0.691

Note: [a] Items were translated to Dutch. [b] Likert-scale ranging from 1 = totally disagree to 5 = totally agree.
